# Peripheral Levels of Renin-Angiotensin System Components Are Associated With Cognitive Performance in Huntington’s Disease

**DOI:** 10.3389/fnins.2020.594945

**Published:** 2020-12-18

**Authors:** Natalia P. Rocha, Courtney Cleary, Gabriela D. Colpo, Erin Furr Stimming, Antonio L. Teixeira

**Affiliations:** ^1^The Mitchell Center for Alzheimer’s Disease and Related Brain Disorders, Department of Neurology, McGovern Medical School, The University of Texas Health Science Center at Houston, Houston, TX, United States; ^2^HDSA Center of Excellence at The University of Texas Health Science Center at Houston, Houston, TX, United States; ^3^Department of Neurology, McGovern Medical School, The University of Texas Health Science Center at Houston, Houston, TX, United States; ^4^Neuropsychiatry Program, Department of Psychiatry and Behavioral Sciences, McGovern Medical School, The University of Texas Health Science Center at Houston, Houston, TX, United States

**Keywords:** Huntington’s disease, renin-angiotensin system, angiotensin, biomarker, cognition

## Abstract

The renin-angiotensin system (RAS) has proven to be involved in the pathophysiology of neurodegenerative diseases, such as Parkinson’s disease (PD) and Alzheimer’s disease (AD), serving as a potential therapeutic target and a disease burden marker. Studies have associated negative clinical outcomes with the activation of the classical RAS arm composed of the angiotensin-converting enzyme (ACE) and angiotensin (Ang) II, while suggested positive outcomes with the activation of the counter-regulatory RAS arm involving ACE2 and Ang-(1–7). Huntington’s disease (HD) shares many pathological and clinical outcomes with AD and PD, but the evidence of direct involvement of RAS components in the pathophysiology of HD is still limited and needs further investigation. Herein, we investigated peripheral levels of the RAS components Ang II, Ang-(1–7), ACE, and ACE2 in controls, premanifest, and manifest HD gene carriers and their relationship with clinical outcomes. Peripheral blood samples were collected via phlebotomy, and plasma concentrations of RAS components were measured by Enzyme-Linked Immunosorbent Assay. Clinical evaluation included a questionnaire about socio-demographic characteristics, motor, and cognitive assessments. Results showed (1) no significant group differences in plasma concentrations of RAS components; (2) positive correlations between ACE2 and Verbal Fluency Test (VFT) scores; and (3) negative correlations between Ang II and Mini–Mental State Examination scores. These results corroborate the proposed balance between the classical (ACE/Ang II) and the counter-regulatory [ACE2/Ang-(1–7)] arms of the RAS, with the former associated with negative clinical outcomes and the latter with positive effects in HD.

## Introduction

The renin-angiotensin system (RAS) is a peptidergic system with endocrine characteristics, well known for its pivotal role in the regulation of water and sodium homeostasis and blood pressure (Paul et al., 2006b). It involves the enzymatic conversion of angiotensinogen to angiotensin (Ang) I by renin. Through the classical pathway, Ang I may be converted to Ang II via the angiotensin-converting enzyme (ACE). Ang II is an active component involved in vasoconstriction, effective circulatory volume maintenance, thirst stimulation, and adrenal gland aldosterone release. Most of Ang II actions are exerted via its binding to angiotensin type 1 receptors (AT_1_; Ferrario and Chappell, 2004). Through an alternative pathway, Ang II may be converted into Ang-(1–7) via ACE2, and this counter-regulatory arm works in opposition to the ACE-AngII-AT_1_ systemic effects. For instance, Ang-(1–7) has cardioprotective effects via reduction of fibrosis and cardiac hypertrophy (Grobe et al., 2007). In addition, Ang-(1–7) has shown beneficial anti-inflammatory effects in hepatic and renal diseases and inhibition of fibrogenesis through the G protein coupled-receptor Mas (Santos et al., 2003; Simoes e Silva et al., 2013; Prestes et al., 2016). The systemic balance between these two RAS branches is of significant physiological importance.

It is now recognized that the RAS is involved in several physiological functions other than cardiovascular and renal homeostasis. In this regard, the RAS has been implicated in several brain functions, including motor control, cognition, and behavior [for a review see Paul et al. (2006a)]. The RAS has proven to be involved in the pathophysiology of neurodegenerative diseases, including Parkinson’s (PD) and Alzheimer’s disease (AD; Munoz et al., 2006; Rocha et al., 2016b, 2018). For instance, Ang II increased neuronal death through activation of oxidative and inflammatory responses (Griendling et al., 2000; Chabrashvili et al., 2003; Rodriguez-Pallares et al., 2008). The neurodegenerative effects of Ang II have been confirmed by experiments with animal models of PD, which have shown a reduction in dopaminergic neuron degeneration with ACE inhibitor treatment (Kurosaki et al., 2005; Munoz et al., 2006). The deleterious effects of Ang II-AT_1_ receptor binding were further confirmed by studies showing the neuroprotective effects of AT_1_ receptor antagonists in animal models of PD (Grammatopoulos et al., 2007; Rey et al., 2007). Although experimental data support a role for the RAS in neurodegeneration, data obtained from human samples are still scarce. We have previously shown that patients with PD have reduced plasma levels of Ang I, Ang II, and Ang-(1–7), which correlated with depressive symptoms (Rocha et al., 2016b). In patients with AD, cerebrospinal fluid (CSF) levels of ACE were reduced in comparison with controls and correlated with reduced amyloid-β (Aβ)_42_ levels, which indicates increased Aβ amyloid accumulation in the brain, a known indicator of AD burden (Rocha et al., 2018). These studies highlight the participation of RAS components in the pathophysiology of neurodegenerative diseases, also indicating possible associations with clinical outcomes. Despite the evidence of RAS-related mechanisms in AD and PD, the role of this system in Huntington’s disease (HD), which shares many neurodegenerative pathways and clinical outcomes with AD and PD (Ross and Tabrizi, 2011), must be explored further.

HD is an autosomal dominant neurodegenerative disease with cognitive, motor, and behavioral symptoms. The HD mutation is described as an expanded CAG trinucleotide repeat in the huntingtin gene (*HTT)* on chromosome 4 encoding huntingtin (HTT), a protein that has proven to be necessary for embryonic development and maintenance. Manifestation age in HD is highly variable and primarily influenced by the length of the CAG trinucleotide repeat with an average age of onset around 45 years (Langbehn et al., 2010). Manifestation or clinical diagnosis of HD has historically been defined as the onset of an “unequivocal presence of an otherwise unexplained extrapyramidal movement disorder (e.g., chorea, dystonia, bradykinesia, rigidity) in a subject at risk for HD” (Hogarth et al., 2005). Subjects with a confirmed expanded CAG trinucleotide repeat but without motor symptoms of HD are classified as premanifest HD gene carriers. HD is regarded as an interesting model to study the pathophysiological mechanisms associated with neurodegeneration because it allows the assessment of patients in a preclinical stage of the disease.

While the cause of HD is known, the mechanisms resulting in neurodegeneration in HD are not fully understood. Similar to AD and PD, inflammation and oxidative stress have been regarded as key-players in HD pathophysiology (Kumar and Ratan, 2016; Rocha et al., 2016a). As well as in other neurodegenerative diseases, the RAS may be an important player in the neurodegenerative outcomes of HD. To the best of our knowledge, no studies have evaluated RAS components in HD gene carriers. Therefore, this study was designed to elucidate whether HD gene carriers present with changes in the RAS. In addition, we sought to explore the potential association between peripheral levels of RAS components and clinical symptoms.

## Materials and Methods

### Subject Evaluation and Biological Sample Collection

Fifty-seven participants (18 premanifest HD gene carriers, 23 manifest HD, and 16 controls) underwent a comprehensive clinical interview and a blood draw. Genetic diagnosis of HD was confirmed by a genotype larger CAG allele ≥36. A movement disorder specialist evaluated all patients, and the clinical diagnosis of HD (manifest HD) was based on the motor signs certainty, i.e., a Diagnostic Confidence Level (DCL) set to 4 in the Unified HD Rating Scale (UHDRS; Ross et al., 2014). All subjects provided written informed consent before admission to the study. The Research Ethics Committees of UTHealth approved this study.

The clinical evaluation included a questionnaire about socio-demographic characteristics, motor, and cognitive assessments. HD gene carriers were subjected to a motor function assessment with the UHDRS. The motor section of the UHDRS assesses motor features of HD with standardized ratings of oculomotor function, dysarthria, chorea, dystonia, gait, and postural stability. It is comprised of 31 items with a 5-point ordinal scale ranging from 0–4, and the total motor score is the sum of all the individual motor ratings, with higher scores indicating more severe motor impairment (Huntington Study Group, 1996). All individuals underwent a brief cognitive examination using the Mini–Mental State Examination (MMSE; Folstein et al., 1975), the Symbol Digit Modalities Test (SDMT), and the Verbal Fluency Test (VFT; Huntington Study Group, 1996). The MMSE is a 30-point questionnaire for cognitive screening, comprising items from different domains such as orientation, attention, memory, and language (Folstein et al., 1975). The VFT and SDMT are part of the UHDRS – cognitive assessment proposed by the Huntington Study Group (Huntington Study Group, 1996). The SDMT is a simple substitution task. Using a reference key, the examinee has 90 s to pair specific numbers with given geometric figures. The score is the number of correct responses achieved in 90 s. The VFT assesses the ability to spontaneously produce words orally within a fixed time span (60 s). For category fluency, words must be produced according to semantic constraints. The measure of performance used is the number of correctly generated words within 60 s (CHDI Foundation, 2011).

Ten milliliters of blood were drawn by venipuncture in vacuum tubes containing heparin on the same day of the clinical assessment. Whole blood samples were used for plasma obtaining within 2 h of having been drawn. These samples were centrifuged at 3,000 *g* for 10 min, 4°C, twice. Plasma was collected and stored at −80°C.

### Biochemical Analysis

For the evaluation of the RAS components, samples were thawed and plasma levels of Ang II, Ang- (1–7), ACE, and ACE2 were measured by Enzyme-Linked Immunosorbent Assay (ELISA), according to the procedures supplied by the manufacturer (MyBioSource, San Diego, CA, United States). Ang-(1–7; MBS084052) and ACE2 (MBS723213) assays employed the quantitative sandwich ELISA technique. Ang II (MBS764273) and ACE (MBS727096) assays were based on the competitive ELISA detection method. Briefly, for the sandwich-ELISA-based assays [Ang-(1–7) and ACE2], standards and samples were pipetted into the wells of a plate that had been pre-coated with antibodies specific for each marker to be analyzed. Any Ang-(1–7)/ACE2 present was bound by the immobilized antibody. After removing any unbound substances, a biotin-conjugated antibody specific for Ang-(1–7)/ACE2 was added to the wells. After washing, avidin conjugated Horseradish Peroxidase (HRP) was added to the wells. Following a wash to remove any unbound avidin-enzyme reagent, a substrate solution (3,3′,5,5′-Tetramethylbenzidine, TMB) was added to the wells, and the color developed in proportion to the amount of Ang-(1–7)/ACE2 bound in the initial step. The color development was stopped by adding sulfuric acid solution and the intensity of the color was measured spectrophotometrically at a wavelength of 450 nm. The procedure for the competitive ELISA assays (Ang II and ACE) was very similar, except for the fact that the sample was incubated together with Ang II-/ACE-HRP conjugate. Ang II/ACE and Ang II-/ACE-HRP conjugates competed for the antibodies binding sites, and the final color intensity was inversely proportional to the Ang II/ACE concentration. The concentrations were calculated based on a standard curve in which the absorbance was plotted against the standard concentration. The sensitivity of the assays was 1.0 pg/mL for ACE and ACE2; 2.0 pg/mL for Ang- (1–7); and <0.094 ng/mL for Ang II.

### Statistical Analysis

Association between dichotomous variables was assessed with the Chi-Square test. All variables were tested for Gaussian distribution by the Shapiro–Wilk normality test. Comparisons between three groups (i.e., controls vs. premanifest HD vs. manifest HD) were made by Kruskal–Wallis or ANOVA tests, according to the non-Gaussian or Gaussian distribution of the variables, respectively. *Post Hoc* analyses were used to determine significant differences between pairs of groups. Spearman’s correlations were performed to examine the relationship between plasma levels of RAS components and: (i) the scores in the clinical scales (UHDRS – Total motor score, SDMT, VFT, and MMSE), and (ii) CAG repeat length. All statistical tests were two-tailed and were performed using a significance level of α = 0.05. Statistical analyses were performed using SPSS software version 26.0 (SPSS Inc., Chicago, IL, United States) and GraphPad Prism version 5.0 (GraphPad Software, Inc., La Jolla, California, United States).

## Results

The demographic and clinical characteristics of the study participants are shown in Table 1. The three groups presented comparable age, sex distribution, and educational level. The groups were significantly different regarding ethnicity. HD gene carriers’ groups (both premanifest and manifest) were mostly comprised of White individuals, while the controls were mostly Hispanics/Latinos. This result is in line with the literature showing higher rates of HD among White/Caucasian populations (Rawlins et al., 2016), and controls were recruited from the local community (Houston, TX, United States), which has a high percentage of Hispanics/Latinos.

**TABLE 1:** Demographic and clinical characteristics of study participants.

**Variable**	**Controls (*N* = 16)**	**Premanifest HD (*N* = 18)**	**Manifest HD (*N* = 21)**	***P* value**
Age in years [mean ± SD (Min – Max)]	48.12 ± 11.02 (32.7–64.0)	44.30 ± 11.34 (24.5–66.6)	49.88 ± 12.64 (21.5–72.1)	0.336^1^
Female sex, N (%)	10 (62.5)	12 (66.7)	14 (66.7)	0.957^2^
Ethnicity, N (%)				
Hispanic or Latino	13 (81.3%)	2 (11.1%)	3 (14.3%)	<0.001^2^
White	3 (18.7%)	16 (88.9%)	17 (81.0%)	
Black or African American	0 (0%)	0 (0%)	1 (4.8%)	
Antihypertensive drugs				
ACE inhibitors	0	2 (11.1%)	1 (4.8%)	
AT_1_ receptor antagonists	2 (12.5%)	2 (11.1%)	1 (4.8%)	N/A^3^
Others*	1 (6.25%)	4 (22.2%)	3 (14.3%)	
Educational Level in years [mean ± SD (median)]	17.28 ± 3.93 (18)	15.17 ± 3.49 (14.5)	15.20 ± 2.07 (15)	0.098^1^
CAG repeats [mean ± SD (median)]	–	41.88 ± 1.80 (41)	44.63 ± 4.14 (43)	0.010^4^
UHDRS – Total motor score [mean ± SD (median)]	–	4.61 ± 4.80 (3)	29.67 ± 12.91 (30)	<0.001^4^
SDMT (total correct) [mean ± SD (median)]	50.88 ± 9.62 (52)^a^	48.00 ± 11.43 (49)^a^	30.55 ± 11.83 (30.5)^b^	<0.001^1^
VFT (category) – number of correct responses in1 min [mean ± SD (median)]	22.38 ± 4.46 (22)^a^	18.39 ± 5.25 (18)^b^	12.55 ± 4.36 (12)^c^	<0.001^1^
MMSE [mean ± SD (median)]	28.13 ± 1.78 (29)^a^	28.33 ± 1.72 (29)^a^	25.85 ± 2.76 (26.5)^b^	0.002^5^

^1^ANOVA followed by Tukey’s multiple comparisons test. Significant differences between groups are indicated by different letters. ^2^Pearson Chi-Square test. ^3^Due to the low expected frequencies, chi-square analysis was not appropriate. ^4^Mann–Whitney test. ^5^Kruskal–Wallis followed by Dunn’s multiple comparisons test. Significant differences between groups are indicated by different letters. *Other antihypertensive drugs include diuretics, calcium channel blockers and beta-adrenergic receptor antagonists. Abbreviations: ACE, angiotensin-converting enzyme; AT1, Angiotensin II type I receptor; HD, Huntington’s disease; MMSE, Mini–Mental State Examination; SD, Standard deviation; SDMT, Symbol Digit Modalities Test; UHDRS, Unified HD Rating Scale; and VFT, Verbal Fluency Test.

**FIGURE 1:**
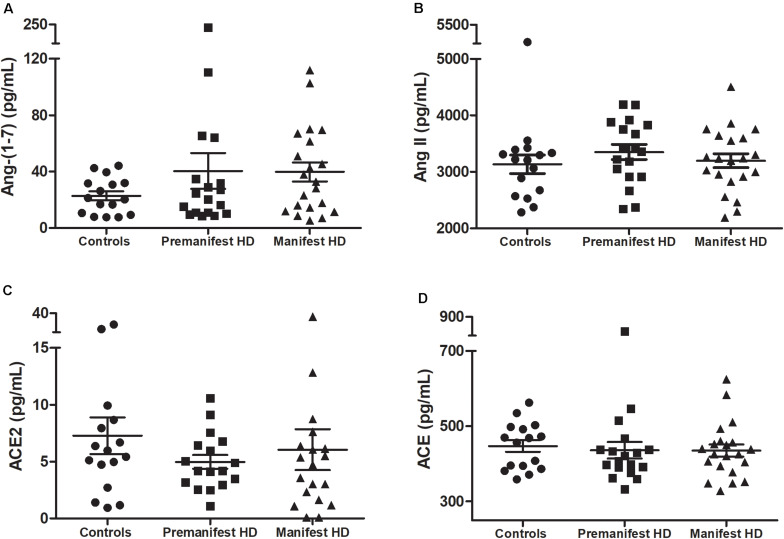
Plasma levels of **(A)** angiotensin-(1–7; Ang-1–7), **(B)** angiotensin II (Ang II), **(C)** ACE2 in controls, premanifest, and manifest Huntington’s disease (HD) individuals and **(D)** angiotensin-converting enzyme (ACE). There were no significant differences between the three groups [Kruskal–Wallis test, as data were determined to not follow a normal distribution]. Horizontal bars indicate mean and standard error of the mean for each set of values.

**FIGURE 2:**
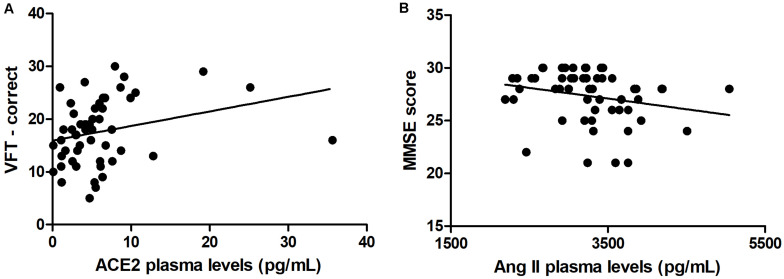
Correlations between renin-angiotensin components and clinical scores. **(A)** Plasma levels of angiotensin-converting enzyme 2 (ACE2) correlated positively with verbal fluency test (VFT) scores. **(B)** Plasma levels of angiotensin II (Ang II) correlated negatively with Mini-Mental State Examination (MMSE) scores. Spearman correlation analyses were performed and a *p*-value < 0.05 was considered significant.

As expected, individuals with manifest HD had worse cognitive performance than premanifest HD gene carriers and controls, as evidenced by the lower scores on the MMSE, SDMT, and VFT. Premanifest HD gene carriers’ scores on the MMSE and SDMT were similar to controls, while the performance of the premanifest group was worse than controls on the VFT (Table 1). Plasma concentrations of RAS components [Ang-(1–7), Ang II, ACE, and ACE2] were determined in controls, premanifest, and manifest HD gene carriers. No significant differences were found when comparing the three groups (Figure 1). There were significant correlations between ACE2 levels and VFT scores (rho = 0.329, *p* = 0.017); and between Ang II levels MMSE scores (rho = -0.341, *p* = 0.012; Figure 2). We did not find any significant correlation between plasma levels of RAS components and SDMT scores, CAG length, or motor score.

## Discussion

This study aimed to evaluate peripheral levels of RAS components in HD gene carriers in comparison with controls, and the potential association between the RAS and clinical outcomes in HD. No significant differences were found between controls, premanifest, and manifest HD gene carriers when evaluating plasma concentrations of Ang-(1–7), Ang II, ACE, and ACE2. However, there were significant correlations between ACE2 levels and VFT scores, and between Ang II levels and MMSE scores.

There are a few studies implicating the RAS in HD pathophysiology. Early studies have found a decrease in ACE activity in brain samples of patients with HD in comparison with controls. The reductions were reported in brain regions involved in HD pathophysiology, such as caudate nucleus (Arregui et al., 1977; Butterworth, 1986), putamen, and globus pallidus (Arregui et al., 1977), in addition to substantia nigra (Arregui et al., 1978). Later, increased ACE activity was reported in the CSF of patients with HD in comparison with controls (Schweisfurth et al., 1987). Decreased ACE activity in the brain may be a result of neuronal death as ACE is expressed by neurons. On the other hand, the increased ACE activity in the CSF can reflect compensatory mechanisms associated with the reduced ACE availability in the central nervous system. The ACE activity can also be influenced by the availability of its substrates and products, and by other RAS components, including ACE2, and angiotensin receptors. Using radioligands, one study described a 35% reduction in AT_1_ receptor levels in the putamen of patients with HD in comparison with controls (Ge and Barnes, 1996). Altogether, these data point to a role of the RAS in HD. Our findings partially corroborate these previous data. While the lack of difference in RAS components between HD gene carriers and controls undermines our hypothesis of the involvement of this system in HD pathophysiology, the observed correlations support it.

The RAS is also known for its role in inflammatory mechanisms. While the classical axis of the RAS (ACE/Ang II/AT_1_ receptor) activates several pathways related to inflammation, the counter-regulatory axis [ACE2/Ang-(1–7)/Mas receptor] exerts anti-inflammatory responses (Rodrigues Prestes et al., 2017). Immune/inflammatory mechanisms are involved in HD pathophysiology (Rocha et al., 2016a) and studies are needed to elucidate how the RAS and inflammatory/immune mechanisms are linked to HD. In this regard, one study reported that anti-AT_1_ receptor antibodies are more frequent in individuals with HD than in controls. The anti-AT_1_ antibody titers correlated with the age of HD onset and disease burden scores and were also linked to smoking and infection. These data suggest a dysfunction of the adaptive immune system in HD, which may be triggered by different stimuli including autoimmune responses, infection, and possibly smoking (Lee et al., 2014).

We found positive correlations between ACE2 and VFT scores, and thus, higher concentrations of ACE2 were associated with better verbal function. Noteworthy, ACE2 activity has been reported to be reduced in AD patients in comparison with controls (Liu et al., 2014; Kehoe et al., 2016). The ratio of Ang II to Ang-(1–7; an indirect estimate of ACE2 activity) was elevated in AD patients, suggesting reduced conversion of Ang II to Ang (1–7; Kehoe et al., 2016). Together, these studies suggest that cognitive decline is associated with reduced RAS counter-regulatory axis components. Our study supports these findings by extrapolating them to HD patients, yet more studies are still necessary to fully determine the beneficial effects of increased ACE2 activity in HD patients. In addition, we found negative correlations between Ang II and MMSE scores, a measure of general cognition. Thus, higher levels of Ang II were associated with worsening cognition. As mentioned before, many studies have revealed the potential neurodegenerative effects of Ang II, and our data is consistent with those reports of negative effects of Ang II (or the classical RAS axis) on cognition.

The deleterious effects of Ang II were further confirmed by an observational study evaluating the effects of anti-hypertensive drugs on HD progression and outcomes. Untreated hypertensive HD patients suffered from more depressive symptoms, worsening cognition, and a more rapid decline in total functional capacity (TFC) and motor function when compared to normotensive and treated hypertensive patients (Steventon et al., 2020). Increasing evidence of the negative effects of untreated hypertension in HD adds value to our proposals of RAS involvement in disease outcomes. Our sample population had a negligible number of participants simultaneously treated with antihypertensive drugs, and thus, no analysis was performed in this regard. To our knowledge, no studies have evaluated the specific effects of RAS-targeted antihypertensive drugs in normotensive HD patients. Yet, the demonstrated protective effects of such medication in hypertensive HD patients is evident and creates an area of scientific curiosity around the role these drugs would play in HD. One study analyzed the use of RAS blockade (RASB) and the incidence of cognitive impairment in AD (Zhuang et al., 2016). Their results demonstrated that RASB was associated with a 35% risk reduction in the incidence of cognitive impairment and 20% risk reduction in the incidence of AD (Zhuang et al., 2016). In addition, the treatment with AT_1_ receptor antagonists attenuates cognitive impairment in a blood pressure-independent manner through reductions in blood-brain barrier permeability in Ang II- and salt-dependent hypertensive patients (Pelisch et al., 2013). These results further emphasize the positive cognitive benefits associated with RAS-targeted antihypertensive drugs in neurodegenerative diseases. More studies must be performed to evaluate their potential protective effect in HD patients.

Our study has several limitations that must be acknowledged. Our sample size was relatively small, preventing us to control for potential confounding factors. The cross-sectional nature of the study also limits inferences of causality. While associations have been made, further research using longitudinal studies following RAS components concentrations throughout the life of HD patients and correlating them with clinical symptoms would be of value to determining the relationship between RAS and HD pathophysiology. In addition, our study did not measure the activity of RAS enzymes (only plasma concentrations) or levels of angiotensin receptors. Therefore, future studies should focus on measures of enzyme activity and receptor levels, which may yield valuable information.

In conclusion, although the RAS components concentrations did not differ among controls, premanifest, and manifest HD gene carriers, there were meaningful correlations between RAS components and cognitive performance in HD. Besides implicating RAS in the pathophysiology of HD, our results corroborate the emerging evidence on the balance between classical and counter-regulatory arms of RAS in neurodegeneration and neuroprotection, respectively.

## Data Availability Statement

The raw data supporting the conclusions of this article will be made available by the authors, without undue reservation.

## Ethics Statement

The studies involving human participants were reviewed and approved by Committee for the Protection of Human Subjects – UTHealth. The patients/participants provided their written informed consent to participate in this study.

## Author Contributions

NR and AT worked on the design and conceptualization of the study. NR, GC, and EF executed the research project. NR and CC designed and executed the data analysis and interpretation. EF and AT reviewed data analysis. NR and CC wrote the first draft of the manuscript. All authors reviewed the manuscript and approved the submitted version.

## Conflict of Interest

The authors declare that the research was conducted in the absence of any commercial or financial relationships that could be construed as a potential conflict of interest.
